# Psychiatric Emergency Bleep Documentation Enhancement Audit

**DOI:** 10.1192/bjo.2024.553

**Published:** 2024-08-01

**Authors:** Anastasija Davidova, Scott Young, Amal Al Sayegh, Jude Halford

**Affiliations:** NHS Lothian, West Lothian, United Kingdom

## Abstract

**Aims:**

West Lothian Psychiatry operates in a district general hospital, fostering a close working relationship between medical and psychiatric practitioners including the Psychiatric 2222 call (akin to medical emergency/cardiac arrest response). No other team like this has been identified in Scotland. Whilst there is a range of scenarios where this is used, there is no ‘gold standard' for defining a psychiatric emergency or how to document these. Preliminary data gathered between August and November 2022 revealed concerns regarding call appropriateness, medical staff proficiency in de-escalation and restraint on medical wards, inadequate handovers, and poor documentation. This prompted a collaborative quality improvement project, undertaken by psychiatric and medical team leaders. Part of this initiative was an audit to improve the documentation of psychiatric emergencies to achieve a 90% compliance rate using a new checklist.

**Methods:**

Cycle 1 of the audit (December 2022 to April 2023) identified patients through the 2222 calls to switchboard (n = 54). TrakCare notes were reviewed to assess call rationale and outcomes, focusing on documentation by the attending psychiatric team. A documentation checklist within the electronic records system was designed and introduced in July 2023, for completion by the junior doctor. Cycle 2 (November 2023 to January 2024, n = 47) aimed to assess improvements by comparing results with the previous cycle.

**Results:**

There was a significant improvement in documentation rates with the checklist (44% to 90%). Indirect enhancements were observed in ward nursing documentation (65% to 83%) and medical ward doctor documentation (39% to 57%). Appropriateness of emergency calls increased from 65% to 74%, with attending doctors' participation in emergencies longer than 10 minutes rising to 68% from 47%. The initial audit revealed a lack of awareness among senior medical staff regarding overnight psychiatric emergency calls, especially in cases of repeated calls for the same individual. The improved documentation played a pivotal role in addressing this issue, facilitating effective information sharing and changes in patient management plans, reducing further emergency calls.

**Conclusion:**

The documentation checklist significantly improved junior doctor documentation, positively impacting patient care and communication among staff. This successful intervention serves as a promising model that can be replicated in other documentation domains. Moreover, this project has set the stage for broader initiatives within a larger Quality Improvement framework. The ongoing efforts are directed towards establishing a shared model for the psychiatric emergency bleep, optimising staffing resources for restraint procedures and improving staff de-escalation skills.

